# Correlating the differences in the receptor binding domain of SARS-CoV-2 spike variants on their interactions with human ACE2 receptor

**DOI:** 10.1038/s41598-023-35070-2

**Published:** 2023-05-30

**Authors:** Gokulnath Mahalingam, Porkizhi Arjunan, Yogapriya Periyasami, Ajay Kumar Dhyani, Nivedita Devaraju, Vignesh Rajendiran, Abisha Crystal Christopher, Ramya Devi KT, Immanuel Dhanasingh, Saravanabhavan Thangavel, Mohankumar Murugesan, Mahesh Moorthy, Alok Srivastava, Srujan Marepally

**Affiliations:** 1 Centre for Stem Cell Research (CSCR) (a Unit of inStem, Bengaluru), CMC Campus, Vellore, Tamil Nadu 632002 India; 2 Department of Biotechnology, SRM Institute of Science and Technology, Chennai, Tamil Nadu India; 3 Centre for Bio-Separation Technology, Vellore Institute of Technology, Vellore, Tamil Nadu 632014 India; 4 Department of Clinical Virology, Christian Medical College, Vellore, Tamil Nadu India

**Keywords:** Translational immunology, Biological techniques, Immunology, Microbiology, Molecular biology

## Abstract

Spike glycoprotein of SARS-CoV-2 variants plays a critical role in infection and transmission through its interaction with human angiotensin converting enzyme 2 (hACE2) receptors. Prior findings using molecular docking and biomolecular studies reported varied findings on the difference in the interactions among the spike variants with the hACE2 receptors. Hence, it is a prerequisite to understand these interactions in a more precise manner. To this end, firstly, we performed ELISA with trimeric spike glycoproteins of SARS-CoV-2 variants including Wuhan Hu-1(Wild), Delta, C.1.2 and Omicron. Further, to study the interactions in a more specific manner by mimicking the natural infection, we developed hACE2 receptors expressing HEK-293T cell line, evaluated their binding efficiencies and competitive binding of spike variants with D614G spike pseudotyped virus. In line with the existing findings, we observed that Omicron had higher binding efficiency compared to Delta in both ELISA and Cellular models. Intriguingly, we found that cellular models could differentiate the subtle differences between the closely related C.1.2 and Delta in their binding to hACE2 receptors. Our study using the cellular model provides a precise method to evaluate the binding interactions between spike sub-lineages to hACE2 receptors.

## Introduction

The severe acute respiratory syndrome coronavirus 2 (SARS-CoV-2) has been continuously evolving into new variants by mutations, resulting in multiple waves of coronavirus disease 2019 (COVID-19) pandemic in human populations worldwide^1^. The entry mechanism of SARS-CoV-2 into host cells is primarily driven by the binding of trimeric spike glycoprotein through receptor-binding motif (RBM) to cognate host receptor (hACE2) followed by receptor-mediated endocytosis^2^. Several new variants harbor single or group of mutations in altered spike proteins interaction with hACE2 which make them more infectious, transmittable, and the dominant strain during COVID-19 pandemics^3^. The first noticeable variant of SARS-CoV-2 was D614G (Pango lineage: B.1, clade 20A) with a single point mutation (substitution of aspartic acid (D) with glycine (G)) in spike protein was identified the beginning of 2020. The D614G showed high affinity to hACE2 by increasing open conformation of RBD (receptor-accessible state) in spike protein then the D614 variant (Wild) with enhanced cellular and host tropism, resulting in more infectious, transmittable, and the dominant pandemic strain that replaced D614 in the mid of 2020^4,5^. Later, several globally dominant strains had been evolved periodically with multiple mutations from D614G strains at a time which replaced its predecessors and became dominant globally. The WHO has classified variants named Alpha, Beta, Gamma, Delta, and Omicron, among others as variants of concern (VOCs)^1^.

The outbreaks of Delta and Omicron variants were dominant across the globe including India by breakthrough infections. Omicron is evolutionarily the most distinct VOC with a high number of mutations (36 coding mutations) in spike protein compared to other VOCs. Due to the higher transmission rate, Omicron has spread rapidly and become the dominant circulating variant globally and has replaced all other previous VOCs^6,7^. Hence, it is critical to understand the binding efficiencies of these variants to the hACE2 receptors to understand potencies of infectivity and transmission rates particularly of dominant Delta and Omicron variants. Multiple computational and experimental studies were attempted to understand the impact of the mutations on binding of Delta and Omicron to hACE2 receptors. However, there were some discrepancies among these studies on evaluating the binding efficiencies of these variants. Several computational studies predicted that the RBD of Omicron showed stronger binding to hACE2 receptors compared to RBD of Wild and Delta respectively^8–14^. Leyun Wu et al.^15^ reported that RBD of Omicron had a weak affinity to hACE2 receptors compared to RBD of Delta, but had similar affinity as RBD of Wild by molecular dynamics (MD) simulations analysis. Similar findings were observed with ELISA, in which RBD of Delta showed higher binding than RBD of Omicron^15,16^. Several attempts were made using multiple biomolecular interaction techniques such as microscale thermophoresis (MST), bio-layer interferometry (BLI), Surface Plasmon Resonance (SPR) to understand affinity between spike variants and hACE2 receptors. Seonghan Kim et al. demonstrated that the RBD of Omicron has a higher affinity towards hACE2 than RBD of Delta by MST analysis. Contrasting results were found in the Maren Schubert et al. study^10,16^. However, BLI approach revealed that both RBDs of Omicron and Delta bind with similar affinities towards hACE2^17^. The inconsistency in evaluating the binding affinities were also observed with SPR analysis as well^18–22^. Hence, a comprehensive strategy including comparing multiple techniques is a prerequisite for a precise understanding of spike variant-hACE2 interactions.

**Figure 1:**
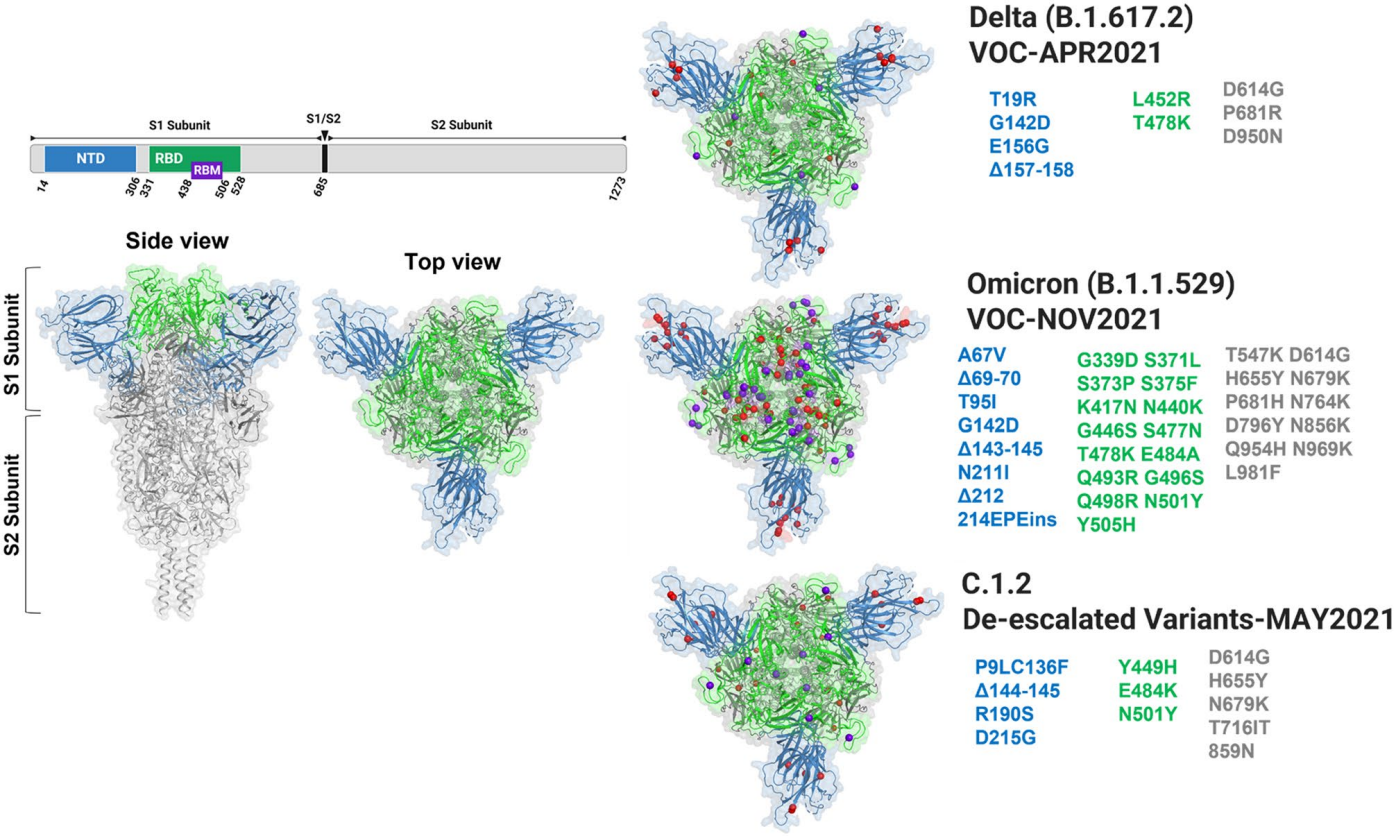
3D crystal structure of ancestral SARS-CoV-2 spike trimer (PDB ID 6XR8)^23^ was generated, highlighting the mutational landscape by purple balls in RBM domain, red balls in NTD (blue), RBD (green) and other (gray) regions of Delta, Omicron and C.1.2 spike trimers using PyMOL software.

Towards understanding the differences in the binding affinities of spike variants including Wild, Delta, Omicron and C.1.2 with hACE2 receptors, in this study, we employed three different methods including ELISA, in vitro flow cytometric binding and competitive binding assay. We also followed three parameters (i) To mimic natural forms of spike protein on SARS-CoV-2 variants, we used trimeric form of spike variants (mutational profile of each variant given in Fig. 1) rather than RBD domain to assess the binding activity towards hACE2 receptors (ii) The differential mutational profiles of spike variants affect the molecular weight of each spike protein of variants. For homogeneity, we evaluated binding efficiencies in molarity, (iii) Binding efficiency of individual spike variants is compared between soluble form (recombinant protein) as well as dimeric (expressed on the cell surface) form of hACE2 receptors.

## Results

### Binding efficiency of trimeric spike variants to soluble hACE2 receptors

Mutation profiles of the spike variants in their native trimeric form affects the proportions of closed to open confirmations of RBD that in turn affect their binding affinities with hACE2 receptors. Wild strain showed more ‘all RBD closed confirmation’ (reduces the affinity) than the other VOC, whereas, Omicron, highly evolved variant showed almost 100% RBD open confirmations^5,24^.

**Figure 2:**
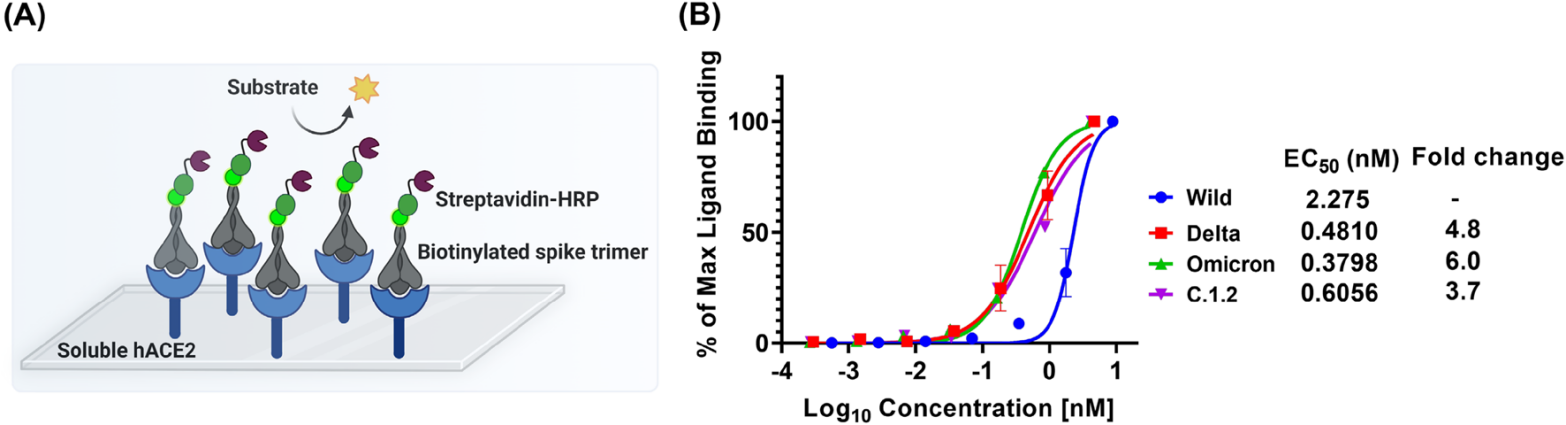
Concentration − response curves of trimeric SARS-CoV-2 spike variants, binding to hACE2 receptor protein. Schematic representation of non-competitive ELISA assay: plate coated with soluble hACE2 receptors and titrated with varying concentrations of biotinylated trimeric spike variants. The level of binding was quantified using streptavidin-HRP conjugate (**A**). The binding curves and EC_50_’s of each spike variants was quantified using ELISA by titrating of biotinylated spike variants to soluble hACE2 coated on the plate and the amount of spike trimer binding were estimated using streptavidin-HRP conjugate (**B**) (Mean ± SEM, N = 2).

Hence, it is important to evaluate the binding affinities of the variants in their native trimeric forms. To this end, firstly, we evaluated the binding efficiencies of trimeric spike variants towards soluble hACE2 receptors using ELISA assay (Fig. 2A). We used non-competitive ELISA for titrating different concentrations of each variant with soluble hACE2 receptors. We calculated the EC_50_ by concentration–response curves for binding of SARS-CoV-2 spike variants to hACE2 using % of maximum ligand binding values. The data showed that Omicron had 6-fold stronger binding towards hACE2 receptors (EC_50_-0.38 nM), than Delta (EC_50_-0.48 nM, 4.8-fold) and C.1.2 (EC_50_-0.61 nM, 3.7-fold) when compared to spike protein of Wild (Fig. 2B). From these findings, Omicron showed superior binding efficiencies towards the hACE2 receptors when compared to Delta and C.1.2 variants.

### Evaluating the binding efficiencies between spike variants using hACE2 receptors expressing cell line

**Figure 3:**
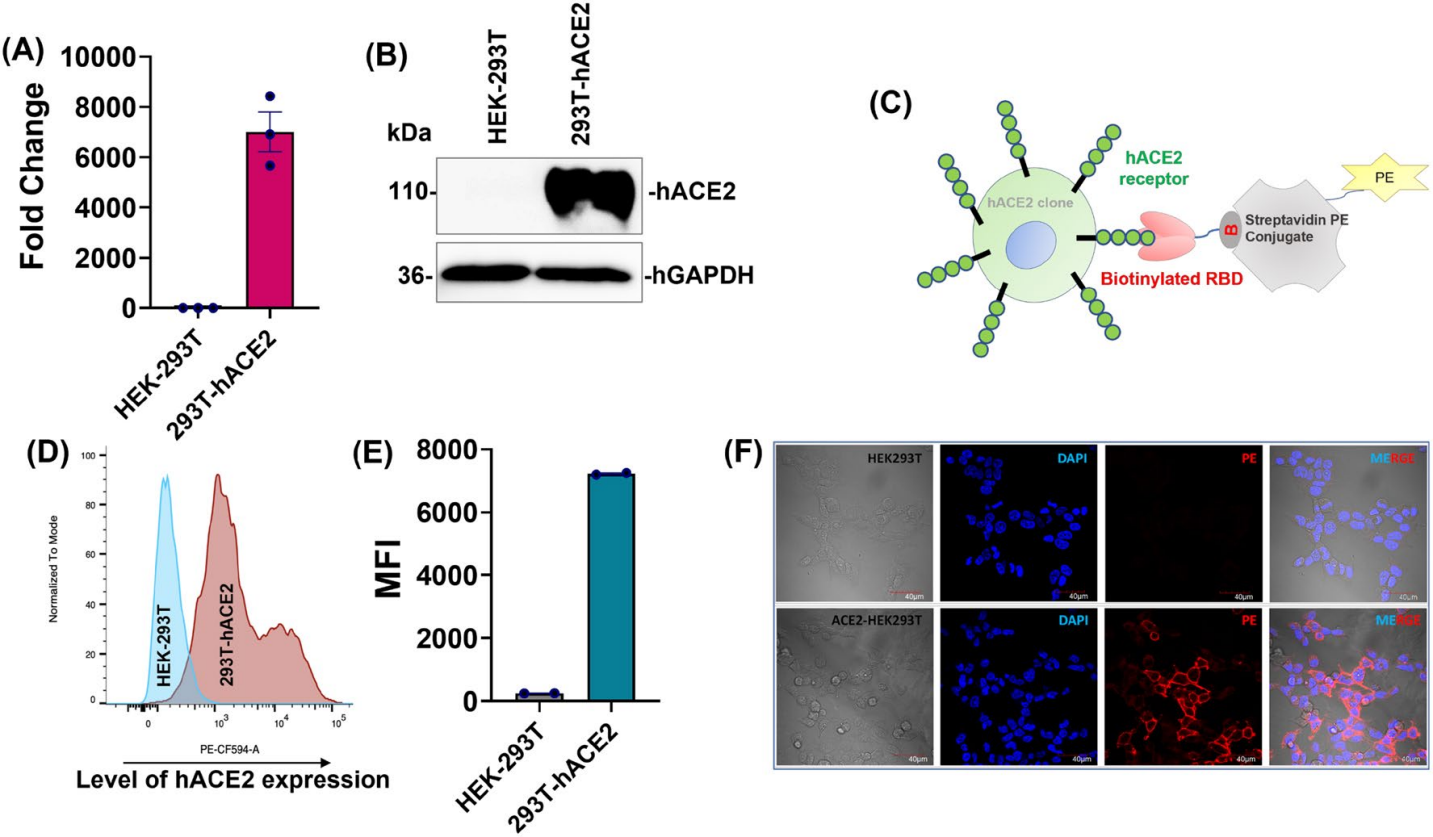
Generation and characterisation of hACE2 receptor stably expressing HEK-293T cell line. The VSV-G lentiviral particles were produced with pLenti-hACE2-P2A-PuroR plasmid and transduced into HEK-293T cells. The stable expressing hACE2 HEK-293T cell line was selected with puromycin. The expression level of hACE2 in 293T-hACE2 cells were analysed by qPCR (**A**) and western blotting (with 20 µg of protein lysates) techniques (**B**). The functional characterization of 293T-hACE2 stable cells were done by RBD-Biotin surface staining. Graphical representation of RBD-biotin surface staining principle (**C**). After RBD-Biotin surface staining, the ancestral SARS-CoV-2 RBD binding hACE2 level on surface of 293T-hACE2 cells were quantified by flow cytometry (**D**, **E**) (MFI-mean florescent intensity) and RBD-hACE2 interaction was visualised by confocal microscopy (**F**).

To closely mimic the natural infection, hACE2 receptors were expressed on the surface of HEK-293T cells and evaluated the binding efficiency of these variants using in vitro flow cytometry binding assay. Firstly, the hACE2 gene was cloned into lenti-backbone and produced lentiviral particles encoded the hACE2 receptors (Fig. S1). These particles were transduced into HEK-293T cells for the generation of stable hACE2 overexpressing cells (293T-hACE2). The expression of hACE2 on 293T-hACE2 cells was confirmed by qPCR (Fig. 3A), which showed overexpression of hACE2 mRNA (~ 7000-fold) and western blot (Figs. 3Band S4), respectively. Further, we confirmed the surface expression of hACE2 receptors on the cells by flow cytometry and fluorescent confocal microscopy (Fig. 3C–F).

**Figure 4:**
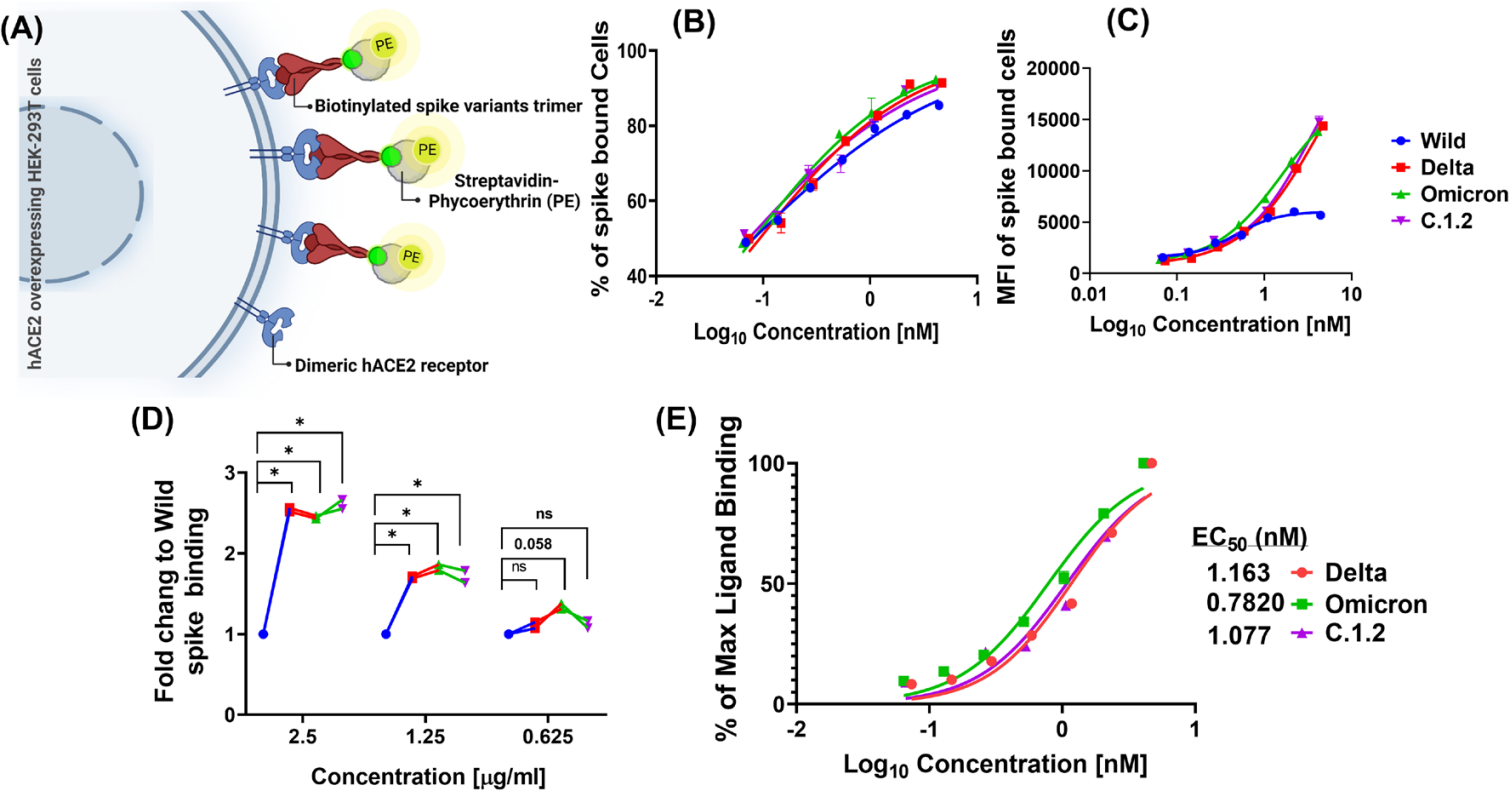
Potency of spike variants affinity to dimeric hACE2 receptors present on surface of the cells. Schematic representation of in vitro flow cytometry binding assay to estimate binding affinity of spike variants (**A**). The 293T-hACE2 cells were surface stained with biotinylated trimeric spike variants at different concentration. Percentage (**B**) and level (MFI) (**C**) of spike trimer bound to cells was quantified using streptavidin-PE conjugate in flow cytometry. The fold change of Delta, Omicron and C.1.2 binding (MFI) related to Wild was calculated at indicated concentration (**D**). Percentage of maximum spike binding was calculated from MFI, plotted against concentration and a non-linear curve fitting was used to estimate the EC_50_ of spike variants (**E**).

Next, we performed in vitro flow cytometry binding assay at different concentrations of trimeric spike variants (Fig. 4A) in 293T-hACE2 cells. Although the percentage of spike trimer binding to hACE2 receptors on the cells was similar among Wild, Omicron, Delta, and C.1.2 spike-stained cells (Figs. 4B and S2), the amounts of spike trimer bound per 293T-hACE2 cells (MFI value) were higher in Omicron, Delta, and C.1.2 spike-stained cells compared to Wild spike-stained cells (Figs. 4C and S3). The fold change of spike binding was calculated by normalizing to Wild spike. We found that around 1.3–2.5-fold increase in their binding efficiencies at higher concentrations of compared to Wild (Fig. 4D). Further, we analysed the EC_50_ by concentration–response curves using % of maximum ligand binding values between Delta, Omicron and C.1.2 spike trimers. The data showed Omicron spike had stronger binding to hACE2 on the surface of the cells (EC_50_-0.78 nM) than Delta (EC_50_-1.2 nM) and C.1.2 spike (EC_50_-1.1 nM), (Fig. 4E). These findings indicate that Omicron shows stronger binding to hACE2 receptors in homodimeric form (expressed on the cells). Moreover, this data revealed that binding efficiencies of both Delta and C.1.2 were similar to hACE2 receptors in its native form on the cells.

### Competing of spike pseudovirus with spike variants, differentiate closely related variants

**Figure 5:**
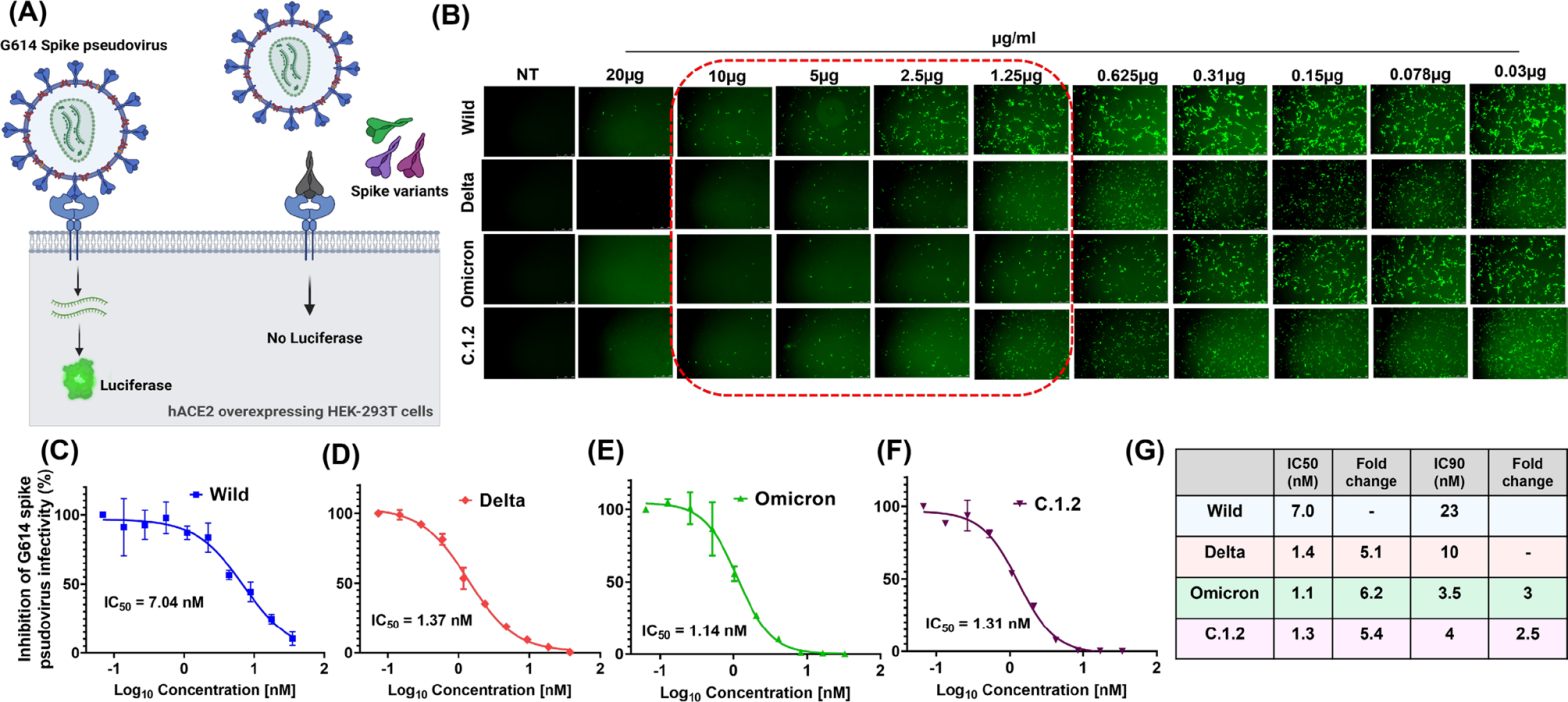
In vitro competitive pseudovirus assay for evaluating the binding affinities of spike variants. Schematic representation of in vitro competitive pseudovirus assay (**A**). The 293T-hACE2 cells was incubated with D614G spike pseudovirus (expressing luciferase and ZsGreen protein) and different concentration of spike variants. After 60–72 h, infectivity of D614G spike pseudovirus was visualized by expression of ZsGreen protein in fluorescent microscopy at different spike variant treatment (**B**). The percentage of D614G spike pseudovirus infectivity was quantified by internalized pseudovirus luciferase activity and inhibitory concentration 50 (IC_50_) of Wild (**C**) Delta (**D**) Omicron (**E**) C.1.2 (**F**) spike proteins were quantified using non-linear curve fitting models. Table of IC_50_ and IC_90_ values of each spike variants (**G**).

To confirm further, we analysed binding efficiencies of spike variants in the presence of D614G spike pseudovirus at different concentrations in 293T-hACE2 cells (Fig. 5A). The pseudovirus has dual reporter genes, ZsGreen for visualization and luciferase for quantification of the infection. Binding of spike variants to the receptor, inhibits the infection of pseudovirus, in turn reduces the expression of reporter genes. Inhibitory concentration (IC) of each spike variant is calculated by the luciferase expression from the pseudovirus infection, in the presence of respective variant. Firstly, inhibition of D614G spike pseudovirus (ZsGreen expression) was visualised in fluorescent microscopy. We observed that Omicron, Delta, and C.1.2 spike proteins inhibited the D614G spike pseudovirus infection more efficiently compared to Wild spike protein in 293T-hACE2 cells (Fig. 5B). Next, we evaluated luciferase expression for highly sensitive detection. Consistent with above two methods, spike protein of Omicron showed lower IC_50_-1.1 nM, compared to Delta (IC_50_-1.4 nM), C.1.2 (1.3 nM) and Wild strain (IC_50_-7 nM) (Fig. 5C–F). Which indicated that Omicron could effectively inhibit the pseudovirus infection at the lowest concentration compared to the other variants and correlated to the superior binding affinity to the hACE2 receptors.The receptor affinity was increased to spike of Omicron (6.2-fold), Delta (5.1-fold), and C.1.2 (5.4-fold) when compared to Wild spike (Fig. 5G). Since mild differences in the affinities of Delta and C.1.2 spike to hACE2 dimer were observed for the IC_50_, we used IC_90_ (inhibit 90% of D614G spike pseudovirus infectivity at concentration of spike variants) to obtain a better comparison. Interestingly, we found that the binding affinities of Omicron (threefold) and C.1.2 (2.5-fold) to hACE2 dimer were higher compared to Delta (Fig. 5G). Overall, the receptor binding studies in cellular model revealed an increasing affinity of spike proteins to the hACE2 receptors from the Omicron > C1.2 > Delta > Wild variant.

### Spike variants showed high immune evasion properties against monoclonal antibody

**Figure 6:**
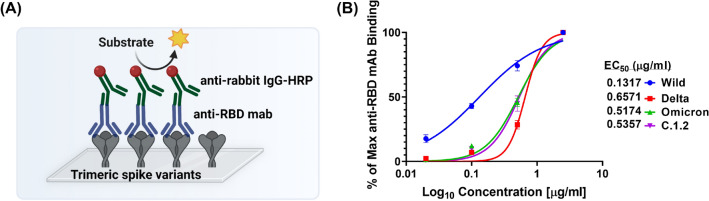
Concentration − response curves of anti-RBD antibody binding to trimeric SARS-CoV-2 spike variants. Schematic representation of ELISA assay to quantify the binding of anti-RBD antibody against trimeric spike variants (**A**). EC_50_ was calculated by titrating of anti-RBD mAbs against trimeric spike variants and level of anti-RBD antibody bound to each spike variants were quantified using anti-rabbit IgG-HRP conjugate (**B**).

Our findings in both ELISA-based assays and cellular models collectively demonstrated that mutational profile on the RBD domain of spike variants increased their binding towards the hACE2 receptors. Spike affinity to hACE2 receptors and immune evasion are responsible for circulation of new variants in human populations. Hence, we also analysed whether the mutations on RBD domain of spike variants affect its antibody binding. To this end, we screened anti-RBD mAbs (raised against the RBD domain of Wild spike) binding to spike variants by ELISA (Fig. 6A). We observed that anti-RBD mAbs showed higher affinity to Wild (EC_50_-0.13 µg/ml), but affinity was significantly reduced to Delta (EC_50_-0.66 µg/ml), Omicron (EC_50_-0.52 µg/ml) and C.1.2 (EC_50_-0.54 µg/ml) variants (Fig. 6B). This study revealed that mutations on spike protein of Delta, C.1.2 and Omicron resulted in immune evasion. Collectively, these studies revealed that Omicron had higher hACE2 receptors binding affinity with immune evasion, resulting in sustaining the current global prevalence.

## Discussion

Understanding the interaction between spike protein variants and the hACE2 receptors is critical for evaluating the infectivity and transmission rates of the VOCs. Most of the studies including molecular modelling studies, biomolecular interaction studies and ELISA were confined to the RBD domain, not to the stable homodimer form of the hACE2 receptors^8–22^. Evaluating the binding energies of trimeric spike to the native form of receptor using molecular modelling simulations is limited by its large size^25^. SPR and BLI-based method involve chemical modifications on the biomolecules for attaching onto the surface of the chip^15^. Recent studies have shown inconsistent data when using certain techniques to analyse the receptor-binding domain (RBD) of spike proteins. This suggests that chemical modifications of the RBD may have an impact on its binding affinity to the receptor and its ability to form a trimer. As a result, these modifications are likely to affect the structure and function of the spike protein differently than the non-modified RBD. Therefore, it is expected that these chemical modifications could lead to discrepancies in binding affinity and spike trimer function. This highlights the need for further exploration of the potential effects of these modifications on the spike protein. Considering the limitations in these techniques, we compared 3 different techniques for evaluation of binding efficiencies. To this end, firstly, we employed non-competitive ELISA, coated with soluble hACE2 receptors, and evaluated the binding efficiencies of trimeric spike from Wild, Delta, C.1.2 and Omicron variants. The trimeric spike of omicron has a 6-fold highest affinity toward soluble hACE2 receptors compared to Delta (4.8-fold), C.1.2 (3.7-fold) than Wild (Fig. 2).

More importantly, recombinantly produced soluble hACE2 (1–740 amino acid residues) are less stable, and spontaneously converted into monomeric form in in vitro condition, hence it needs to be stabilized with dimeric FC domain^26,27^. Moreover, recent findings reported that dimeric forms of hACE2 either in soluble or on the cell surface enhanced the spike protein binding than monomeric forms and disruption in the dimerization significantly affected the interactions^26–29^. Hence, it is ideal to evaluate the receptor binding interactions of spike variants in a close-to-natural phenomenon.

Evaluating and correlating the binding efficiencies of spike variants in cellular models is still an elusive. To this end, we studied the binding efficiencies of spike proteins with hACE2 receptors, expressed as homodimeric form on the surface of the cells using biotinylated trimeric spike variants followed by fluorescently conjugated streptavidin binding and evaluated the fluorescent intensity in flow cytometry. Consistence with ELISA using soluble hACE2 receptors data, the affinity of trimeric spike of Omicron showed higher affinity towards hACE2 receptors on the cells than Delta and C.1.2., but the affinity of a trimeric spike of C.1.2 variants showed slightly higher than Delta variant towards hACE2 receptors on the cell surface (Fig. 4E). We further compared these interactions in a competitive binding experiment by evaluating the levels of infectivity of SARS-CoV-2 D614G spike pseudovirus in presence of trimeric spike variants. D614G is the first single point mutation in spike glycoprotein that contributes to an increased binding of SARS-CoV-2 virus to hACE2 receptors and results in higher rates of infectivity^30^. All the variants including Delta, C.1.2., and Omicron are evolved from D614G lineage (Fig. 1). Hence, we used D614G spike pseudovirus for studying the binding interactions. Competitive binding with the pseudovirus revealed the trimeric spike of Omicron (3-fold) and C.1.2 (2.5-fold) variant was found to be higher affinity to hACE2 receptor when compared to Delta variant (Fig. 5G).

Both computational studies and in vitro studies demonstrated that N501Y mutation on spike protein enhanced the binding affinity towards hACE2 receptors^31–34^. In concurrence, we observed similar binding affinity of N501Y mutation containing variants including Omicron and C.1.2 showed increased binding to hACE2 receptors than Delta (does not containing N501Y mutations) in the cellular model. Rapidly expanding variant (Omicron) showed higher affinity than the C.1.2., possibly because of additional mutations such as S477N and Q498R in Omicron variants, known to strengthen the affinity of hACE2 as well as cross-species ACE2 receptors^32^.

This study provides a precise approach for evaluating the binding strength of spike to cognate receptors. Using two different approaches involving cellular models, we identified the subtle differences between the closely related C.1.2 and Delta in their binding to hACE2. Overall, this approach would be helpful in evaluating the binding efficiencies of emerging variants to understand the virus-host interactions, disease progression and in screening of potent therapeutic molecules.

## Materials and methods

### Cloning of hACE2 gene into lentiviral transfer plasmid

The hACE2 ORF sequence amplified from the hACE2 vector (Addgene, #1786) by high fidelity Q5 polymerase PCR using primer set (Table S1) and cloned purified hACE2 gene fragment into NheI and BamHI digested fragment of pLenti backbone (Addgene, #112675) by Gibson assembly. The hACE2 clones (pLenti-hACE-P2A-Puro) were confirmed by PCR and restriction digestion with NheI and BamHI enzymes.

### ELISA for hACE2 and spike variants binding

The hACE2 protein (SinoBiological, 10108-H08H) was coated on high binding 96 well Plate (Biomat, MT01F4-HB8) at 0.1 μg per well concentration in phosphate-buffered saline (PBS, pH-7.4) overnight at 4 °C. Later, plate was washed 3× with washing buffer (PBS-T, 0.05% Tween 20 in PBS) and blocked with 3% BSA in PBS-T for 2 h at RT. After 3 × washing, incubated with 100 μl of the biotinylated trimeric spike protein of Wild, Delta, C.1.2, Omicron variants (ACRO Biosystems) at different concentrations (fivefold dilution in FC buffer from 5 to 0.039 µg/ml) in diluent buffer (1% BSA in PBS-T) to hACE2 coated wells for 1 h at 37 °C. After removing unbound with 5X washing, all wells were incubated with 100 μl of streptavidin-HRP reagent (Southern Biotech) diluted 1/5000 in 1% BSA in PBS-T for 1 h at 37 °C. After 5X washing, 3, 3′, 5, 5′-Tetramethylbenzidine (TMB) substrate (Bioponda Diagnostics, TMB-S-005) was added and stopped after 5 min with stop solution (Bioponda Diagnostics, STP-001). Each well was read for optical density (OD) at 450 nm in i3x plate reader (Molecular Devices). OD of samples was subtracted from OD of blank (Diluent buffer only). The percentage of maximum spike binding was calculated by the following formula: [OD of concentrations/OD of highest concentration of spike variants)] × 100%. The Half maximal effective concentration (EC_50_) of each variant was calculated by agonist versus normalized response–variable slope model.

### Generation of stably expressing hACE2 HEK-293T (293T-hACE2) Cells

The hACE-P2A-Puro gene encoding VSV-G lentiviral particles were produced by second generation lentiviral plasmids (Addgene) and transfected the pLenti-hACE-P2A-Puro, psPAX2 and pMD2.G plasmids at 2:1:1 ratio into HEK-293T cells (NCCS, Pune, India) at 75–80% cells confluency using Lipofectamine™ 3000 transfection reagent (LF-3000, Invitrogen). After 60 h post transfection, the lentiviral particles were purified using lentivirus concentrator (Takara) in supernatant and reconstituted in 40 µl DMEM media and stored at − 80 °C. Later, transduced 40 µl of purified lentiviral particles to 0.5 million HEK-293T cells in D10 media supplemented with 5 mM HEPES buffer and polybrene at 6 μg/ml concentration in a six-well plate. After 48 h post transduction, hACE2 stable clones were established for 5 passages using puromycin at 2 μg/ml concentration in the D10 medium. The expression of hACE2 in selected clones was confirmed by qPCR, western blot and RBD surface staining followed by flow cytometry analysis and confocal microscopy.

### qRT-PCR analysis

We used the protocol for qRT-PCR as described^35^. Total RNA was isolated from one million cells using RNA iso-plus reagent as per manufacture protocol (Takara) from HEK-293T and 293T-hACE2 cells. 500 ng of total RNAs are converted into cDNA using cDNA synthesis kit (Takara). Syber green-based qPCR was performed using primer sets (Table S1) with 50 ng of cDNA from each sample in the QuanStudio-6 qPCR system (Applied Biosystems) with thermocycler condition: Stage1: 95 °C for 30 s, Stage:2 followed by 40 cycles of 95 °C for 5 s at 60 °C for 30 s in Real-time PCR system. The Ct value of hACE2 normalized with the Ct value of internal control (GAPDH gene expression) in each sample and fold change was calculated by the 2 − ΔΔCT method.

### Western blot

The protein lysates were prepared from HEK-293T and 293T-hACE2 cells using RIPA buffer (Thermos scientific, VH310061) with protease inhibitor (Medchem Express, HY-K0010) and estimated protein concentration of each sample by the Bradford assay using BSA standards. 20 μg of protein lysates were resolved in 10% SDS-PAGE gel and transferred onto a PVDF membrane (WH3135834). After blocking with 5% non-fat dry milk powder in TBST, the blot was incubated with hACE2 antibody (Thermo Scientific, MA5-32307, and dilution at 1 in 2000 in blocking buffer) and followed by goat anti-rabbit IgG H&L-HRP (Invitrogen, ab205718, dilution of 1 in 10,000 in blocking buffer). The blot was visualized for the hACE2 band using a chemiluminescence substrate in Chem-Doc imaging system (Bio-Rad). For internal control, the blot was further stripped and probed for GAPDH (Biorad, MCA4740, dilution at 1 in 2000 in blocking buffer) with anti-mouse IgG antibody H&L-HRP (VectorLab, PI-2000, dilution at 1 in 10,000 in blocking buffer), and visualization of bands in the blot was performed.

### Flow cytometry

2.5 × 10^5^ HEK-293T and 293T-hACE2 cells resuspend in 100 µl of FC buffer (2% heat in-activated FBS and 0.05% NaN3 in PBS) containing 1 µg/ml biotinylated RBD ligand (SinoBiological, 40592-V08H-B) for 30 min at 4 °C in FACS tube. The cells were washed twice with 1 ml of FC buffer and centrifuged at 1000 rpm for 5 min. The supernatant was discarded and then incubated with 100 µl of Streptavidin-PE-CF549 (BD Bioscience) conjugate 1/100 dilution in FC buffer for 30 min. After 2X washing, the cells were analysed using BD Celesta and mean fluorescent intensity (MFI) of each sample was analysed by FlowJo software.

### Fluorescence confocal microscopy

2 × 10^5^ 293T-hACE2 cells were grown on glass bottom confocal dish (35 mm, Ibidi) for 24 h and fixed cells on dish using 300 µl cold 4% paraformaldehyde for 15 min. After 3X washing with 2 ml of cold wash buffer (4% BSA in PBS), incubated with 300 µl of 1 µg/ml of the biotinylated RBD ligand for 45 min at 4 °C and followed by staining with streptavidin-PE-CF549 conjugate (BD Bioscience) at 1/100 dilution for 45 min at 4 °C and washed three times with wash buffer. The cells were stained with 300 µl of DAPI (10 µg/ml) for 15 min at room temperature and washed for one time followed by the addition of two drops of anti-fade (VectorLab, H-1000-10) and observed under a confocal microscope.

### In vitro flow cytometry binding assay

1 × 10^5^ 293T-hACE2 cells in 100 µl FC buffer were incubated with different concentrations (twofold dilution in FC buffer from 2.5 to 0.039 µg/ml) of the biotinylated trimeric spike protein of Wild, Delta, C.1.2, Omicron variants for 30 min at 4 °C. The cells were washed twice with FC buffer and incubated with streptavidin − PE conjugate (BD bioscience, 349023) for 30 min at 4 °C. Cells were washed with PBS, centrifuged, and reconstituted in 100 µl of FC buffer. The stained cells were analysed in flow cytometry BD Celesta. Level of spike proteins binding to hACE2 receptors on cells was evaluated using Flow Jo software. The percentage of maximum spike binding for each variant was calculated by MFI using the following formula: [MFI of each concentration/MFI of highest concentration of spike variants)] × 100%. The Half maximal effective concentration (EC_50_) of each variant was calculated by agonist versus normalized response–variable slope model.

### Production of D614G spike pseudotyped Lentiviruses

D614G spike pseudotyped Lentiviral particles were produced as per the protocol described in^30^. Briefly, HEK-293T cells at 50-70% confluent in a 100 mm plate was transfected with BEI lentiviral plasmids at a concentration of 5 μg pLuciferase-IRES-ZsGreen, 1.1 μg pHMD-Hgpm2, 1.1 μg plasmid pRC-CMV-Rev1b, 1.1 μg pHDM-tat1b and 1.7 μg pD614G spike protein in 10 ml of D10 growth media (10% FBS in DMEM media) using Lipo-DOH transfection reagents. At 18–24 h post-transfection, media was replenished with 10 ml of D10 media and incubated at 37 °C for 60 h. Later, D614G spike pseudoviruses were harvested by collecting the supernatant from plate, centrifuged, and filtered by using a 0.45 µm PVDF low protein-binding filter, and stored at − 80 °C for further analysis.

### In vitro spike pseudovirus competitive assay

D614G spike pseudovirus competitive assay was performed with a modification of pseudovirus neutralization assay as described^30^. Briefly, the 293T-hACE2 cells were seeded (1.25 × 10^4^ per well) in 0.1 mg/ml poly-l-lysine-coated 96-well plate. After 16 h, cells were incubated with 100 µL of D614G spike-pseudovirus (~ 2–4 × 10^6^ RLU per mL of D10 media) along with the different concentrations of trimeric spike protein of Wild, Delta, C.1.2, Omicron (20–0.039 µg/ml) at 37 °C in CO_2_ incubator. At 60–72 h post transduction, infectivity of D614G spike-pseudovirus was visualized by expression of ZsGreen level under fluorescence microscopy (Leica), and quantified infectivity by luciferase activity using Steady-Glo® luciferase reagent (Promega, E2510). From Relative Luciferase Unit (RLU) values, the percentage of maximum D614G spike pseudovirus infectivity for each variant at different concentration was calculated by the following formula: [RLU of each concentration/RLU of highest concentration of spike variants)] × 100%. Half-maximal inhibitory concentration (IC_50_) of each variant was calculated using [Inhibitor] versus response–variable slope (four parameters).

### ELISA for anti-RBD antibody and spike variants interaction

The trimeric spike protein of Wild, Delta, C.1.2, Omicron variants were coated on high binding 96 well plate (Biomat, MT01F4-HB8) at 0.1 μg per well using phosphate-buffered saline (PBS, pH-7.4) overnight at 4 °C. Blocked with 3% BSA in PBS-T for 2 h at room temperature and incubated with 100 μl of anti-RBD monoclonal antibodies (Invitrogen, P06DHuRb, twofold diluted in 1% BSA in DPBS-T from 2.5 to 0.02 µg/ml concentration) to spike variants coated wells for 1 h at 37 °C. All wells were washed and incubated 100 μl of goat anti-rabbit IgG-HRP antibody at dilution of 1 in 5000 in blocking buffer. The level of anti-RBD bound to each spike variants were estimated by 3, 3′, 5, 5′-Tetramethylbenzidine (TMB) substrate. The level of binding of IgGs in each sera sample to spike variants were quantified by measuring the optical density (OD) at 450 nm in i3x plate reader (Molecular Devices, San Jose, CA). OD of each sera sample for spike variants was subtracted to respective spike variants blank OD (without sera). The percentage of maximum anti-RBD antibody binding was calculated by the following formula: [OD of concentrations/OD of highest concentration of spike variants)] × 100%. The Half maximal effective concentration (EC_50_) of each variant was calculated by agonist versus normalized response–variable slope model.

### Statistical analysis

The statistical significance and visualization of data were generated in GraphPad Prism version 8.0. Statistical significance of spike variant binding to hACE2 receptors was analysed by unpaired t-test with Welch's correction. Equal or less than p values, of *p < 0.05, **p < 0.01, considered as significant and ns = non-significant.

## Supplementary Information

Supplementary Information.

## Data Availability

Original data is included in the article. Additional data can be accessed from the corresponding author on the request.
