# Supplementary material for: Correlating the differences in the receptor binding domain of SARS-CoV-2 spike variants on their interactions with human ACE2 receptor
Source: Sci Rep. 2023 May 30;13:8743. doi: 10.1038/s41598-023-35070-2 (PMC10227802; doi:10.1038/s41598-023-35070-2)
Supplement: Supplementary file 1 — Supplementary Information. [file 41598_2023_35070_MOESM1_ESM.pdf]

Correlating the differences in the receptor binding domain of SARS-CoV-2 spike variants on their interactions with human ACE2 receptor

Gokulnath Mahalingam[1], *Porkizhi Arjunan*[1], *Yogapriya Periyasami*[1], *Ajay Kumar Dhyani*[1], *Nivedita Devaraju*[1], *Vignesh Rajendiran*[1], *Abisha Crystal Christopher*[1], *Ramya Devi KT*[2], *Immanuel Dhanasingh*[3], *Saravanabhavan Thangavel*[1], *Mohankumar Murugesan*[1], *Mahesh Moorthy*[4], *Alok Srivastava*[1], *Srujan Marepally*[1]*

1. Centre for Stem Cell Research (CSCR) (a unit of inStem, Bengaluru), CMC Campus, Vellore, 632002, TN, India

2. Department of Biotechnology, SRM Institute of Science and Technology, Tamil Nadu, India

3. Centre for Bio-Separation Technology, Vellore Institute of Technology, Vellore, Tamil Nadu, 632014, India.

4. Department of Clinical Virology, Christian Medical College, Vellore, Tamil Nadu, India

Correspondence: Dr. Srujan Marepally, Ph.D., CSCR-Vellore, srujankm@cmcvellore.ac.in

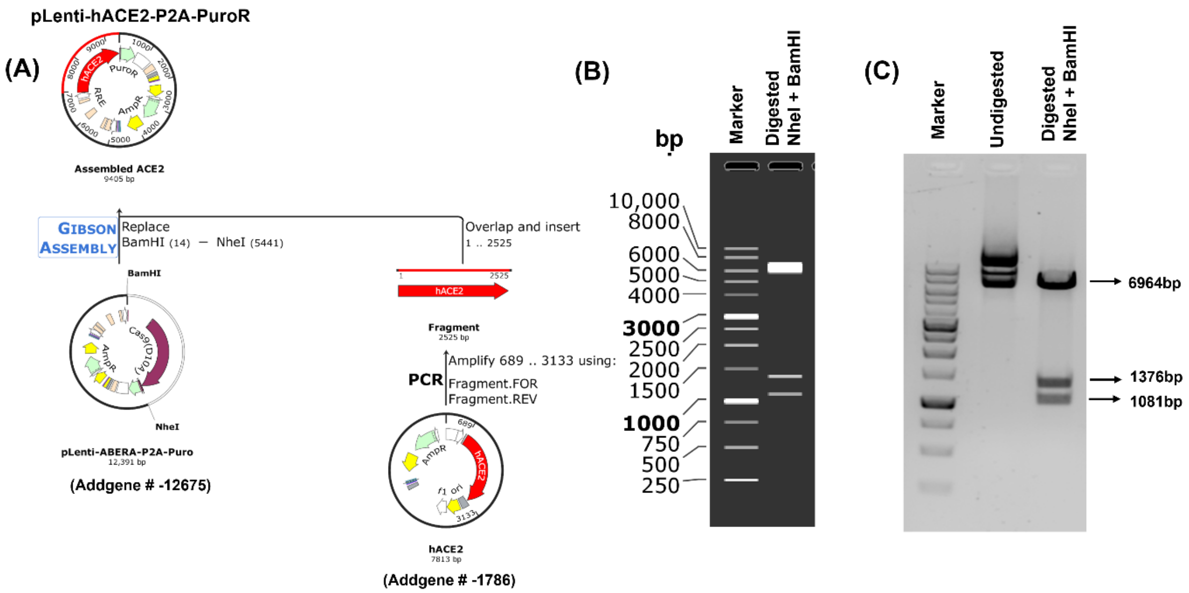

Figure S1: Cloning of hACE gene and characterization. Overview of molecular cloning of hACE2 gene into lentiviral plasmid by Gibbson assembly (A). The fragmentation of pLenti-hACE2-P2A-PuroR plasmid by restriction digestion (NheI and BamHI enzyme) was predicted insilico by Snapgene software (B) and confirmed by in vitro digestion (C).

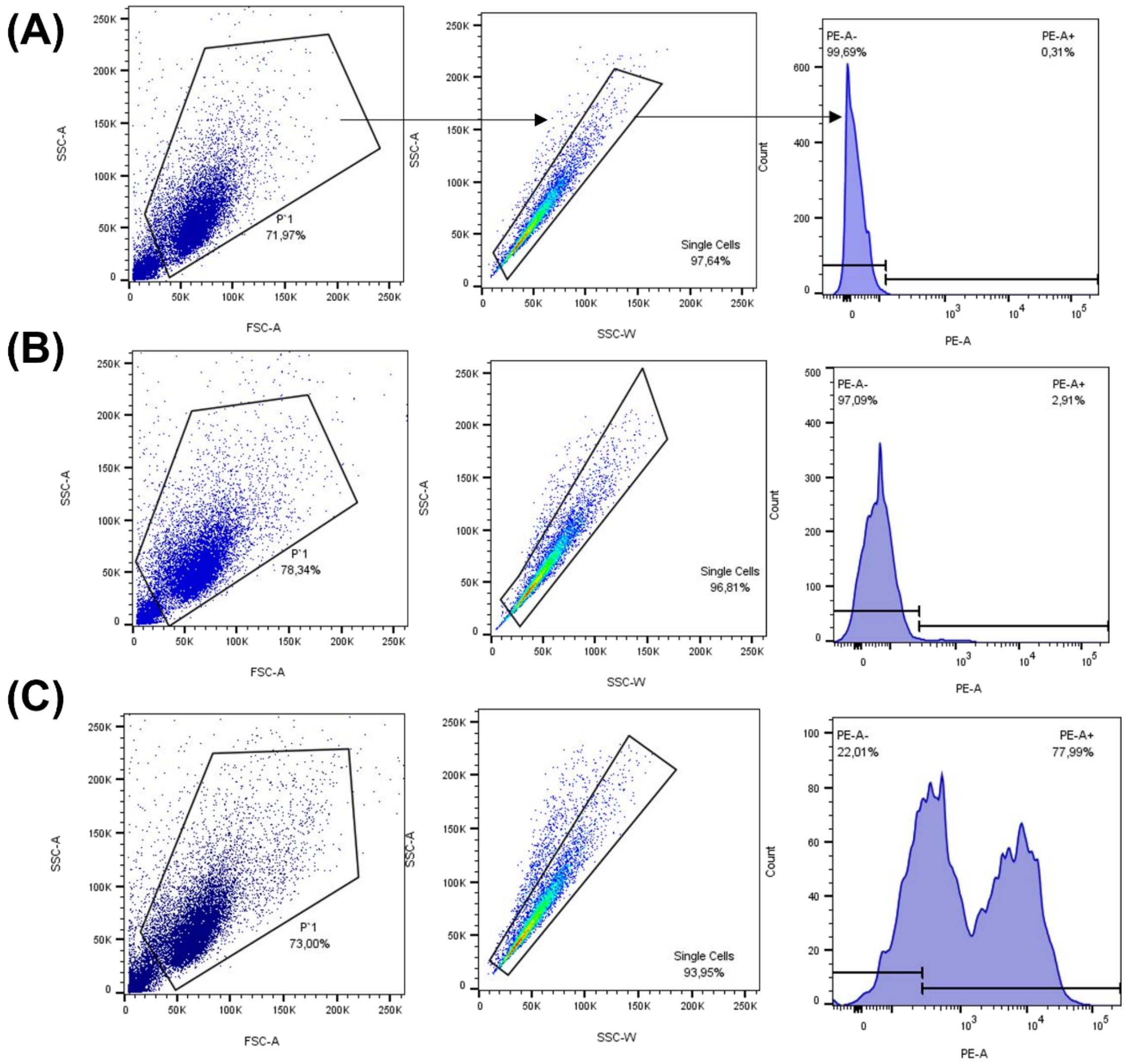

Figure S2: Gating strategy of Flow cytometric evaluation. P1 Shows Population of the 293T-hACE2 cells. To discriminate the doublets, single cells were gated from P1 Population. PE+ve cells were gated from the singlet population. Unstained (A), Histograms for Isotype controls for Streptavidin-PE (B), Wild spike bound cells (C). The PE positive was gated against isotype as a control.

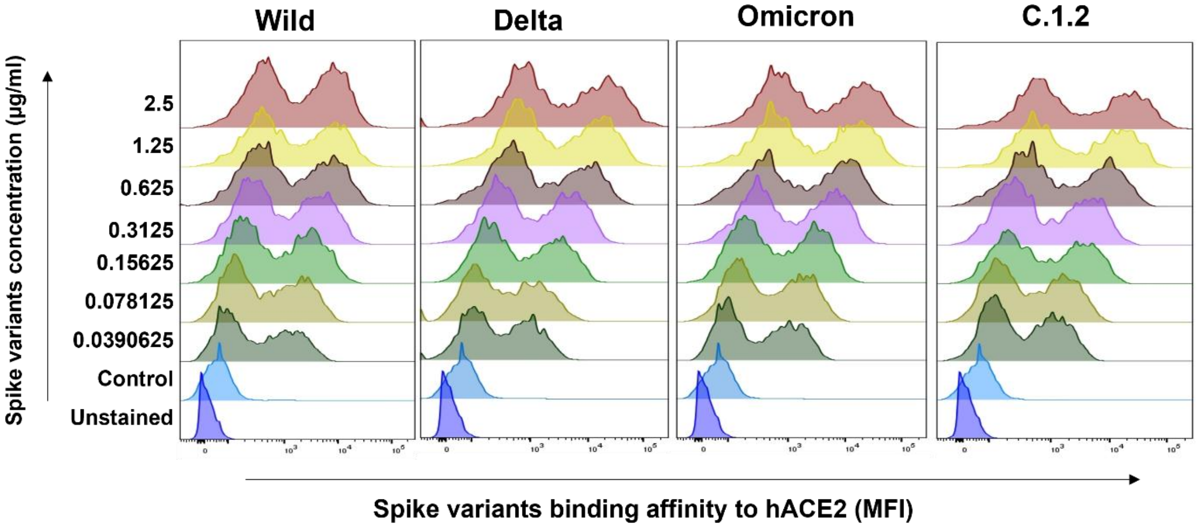

Figure S3. Histogram plots show the level of spike variants binding to dimeric hACE2 receptor present on surface of the cells at different concentrations, generated by FlowJo software. Unstained-cells only, Control-cells + streptavidin−PE conjugate.

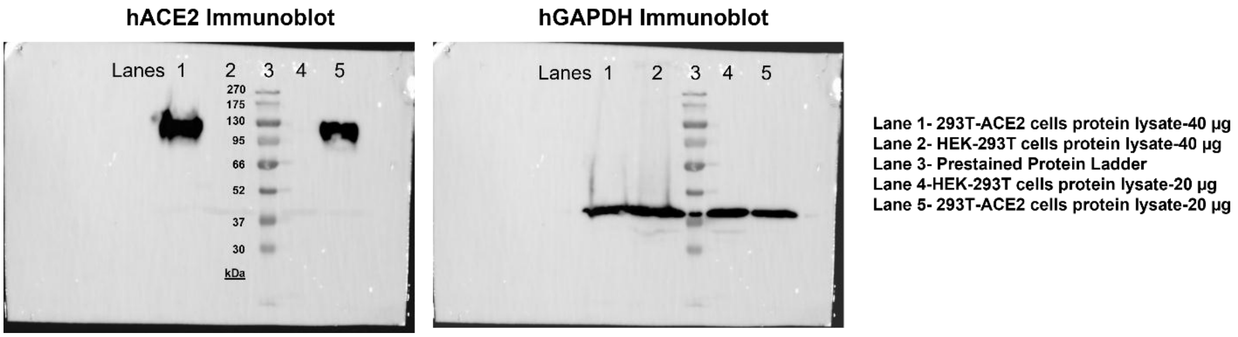

Figure S4. Whole blot images of western blot, performed with 20 µg and 40 µg of protein lysate from HEK-293T and 293T-ACE2 cell lines, and prestained protein ladder (Bio-Helix, #PMB01-0500).

Table S1. Primer List

Primer name	Primer sequence 5’- 3’ direction
**Human ACE2 cloning**	F-CCAGAACACAGGTGTCGTGACGCGGGATCCGCCACCATGTCAAGCTCTTCCTGGCTCC
	R- GAAGTTGGTGGCGCCGCTGCCGCTAGCAAAGGAGGTCTGAACATCATCAGTGTTTTG
**Human ACE2**	F- GGGATCAGAGATCGGAAGAAGAAA
	R- AGGAGGTCTGAACATCATCAGTG
**Human GAPDH**	F- CTGGGCTACACTGAGCACC
	R- AAGTGGTCGTTGAGGGCAATG
